# Acute adverse events of ultra-hypofractionated whole-breast irradiation after breast-conserving surgery for early breast cancer in Japan: an interim analysis of the multi-institutional phase II UPBEAT study

**DOI:** 10.1007/s12282-024-01577-3

**Published:** 2024-04-12

**Authors:** Peter J. K. Tokuda, Takamasa Mitsuyoshi, Yuka Ono, Takahiro Kishi, Yoshiharu Negoro, Setsuko Okumura, Itaru Ikeda, Takashi Sakamoto, Yumi Kokubo, Ryo Ashida, Toshiyuki Imagumbai, Mikiko Yamashita, Hiroaki Tanabe, Sayaka Takebe, Mariko Tokiwa, Eiji Suzuki, Chikako Yamauchi, Michio Yoshimura, Takashi Mizowaki, Masaki Kokubo, the Kyoto Radiation Oncology Study Group

**Affiliations:** 1 Department of Radiation Oncology, Kobe City Medical Center General Hospital, 2-1-1, Minatojima Minamimachi, Chuo-ku, Kobe, Hyogo 650-0047 Japan; 2 Department of Radiation Oncology and Image-Applied Therapy, Graduate School of Medicine, Kyoto University, Kyoto, Kyoto Japan; 3 Department of Radiation Therapy, Osaka Red Cross Hospital, Osaka, Osaka Japan; 4 Department of Radiation Oncology, Japanese Red Cross Wakayama Medical Center, Wakayama, Wakayama Japan; 5 Department of Radiation Oncology, Hyogo Prefectural Amagasaki General Medical Center, Amagasaki, Hyogo Japan; 6 Department of Radiation Oncology, Shiga General Hospital, Moriyama, Shiga Japan; 7 Department of Radiation Oncology, Kyoto-Katsura Hospital, Kyoto, Kyoto Japan; 8 Department of Radiological Technology, Kobe City Medical Center General Hospital, Kobe, Hyogo Japan; 9 Department of Breast Surgery, Kobe City Medical Center General Hospital, Kobe, Hyogo Japan

**Keywords:** Breast cancer, Interim analysis, Phase II study, Ultra-hypofractionated radiation therapy, Whole-breast irradiation

## Abstract

**Background:**

The applicability of ultra-hypofractionated (ultra-HF) whole-breast irradiation (WBI) remains unknown in Japanese women. This study aimed to evaluate the safety and efficacy of this approach among Japanese women and report the results of an interim analysis performed to assess acute adverse events (AEs) and determine whether it was safe to continue this study.

**Methods:**

We enrolled Japanese women with invasive breast cancer or ductal carcinoma in situ who had undergone breast-conserving surgery, were aged ≥ 40 years, had pathological stages of Tis–T3 N0–N1, and had negative surgical margins. Ultra-HF-WBI was delivered at 26 Gy in five fractions over one week. When the number of enrolled patients reached 28, patient registration was paused for three months. The endpoint of the interim analysis was the proportion of acute AEs of grade ≥ 2 (Common Terminology Criteria for Adverse Events v5.0) within three months.

**Results:**

Of the 28 patients enrolled from seven institutes, 26 received ultra-HF-WBI, and 2 were excluded due to postoperative infections. No AEs of grade ≥ 3 occurred. One patient (4%) experienced grade 2 radiation dermatitis, and 18 (69%) had grade 1 radiation dermatitis. The other acute grade 1 AEs experienced were skin hyperpigmentation (n = 10, 38%); breast pain (n = 4, 15%); superficial soft tissue fibrosis (n = 3, 12%); and fatigue (n = 1, 4%). No other acute AEs of grade ≥ 2 were detected.

**Conclusions:**

Acute AEs following ultra-HF-WBI were within acceptable limits among Japanese women, indicating that the continuation of the study was appropriate.

**Supplementary Information:**

The online version contains supplementary material available at 10.1007/s12282-024-01577-3.

## Introduction

Breast-conserving surgery (BCS) followed by whole-breast irradiation (WBI) is an established treatment for early breast cancer. According to a previous meta-analysis, WBI following BCS reduced the risk of recurrence within 10 years by 15.7% (35.0% vs. 19.3%, P < 0.00001) [1]. The conventionally fractionated (CF) WBI consists of 25 daily fractions of 2 Gy each, delivered across approximately five weeks. In recent years, hypofractionated (HF) WBI, which delivers radiation in 15–16 treatments, represents another fractionation regimen that has been recommended in international consensus guidelines [2, 3].

HF-WBI has become more common in Japan following the results of a multicenter prospective trial among Japanese women who underwent HF-WBI after BCS (the JCOG0906 study) [4, 5]. According to a nationwide survey in 2016, 38% (111/293) of the participating facilities used HF regimens for WBI [6]. HF-WBI is also recommended by the Japanese Breast Cancer Society [7].

Ultra-HF-WBI consists of fewer fractions but delivers larger daily doses than both CF- and HF-WBI, with the aim of reducing the overall treatment duration. The UK FAST-Forward trial proved the non-inferiority of ultra-HF-WBI (26 Gy in five fractions over one week) to the standard 40 Gy in 15 fractions over three weeks, in terms of ipsilateral breast tumor relapse and clinician-assessed effects on normal tissues [8, 9]. In other countries, primarily in Europe, ultra-HF-WBI is also under investigation in clinical trials and is progressing toward implementation [10, 11].

However, ultra-HF-WBI is not yet clinically practiced in Japan, and its safety in Asian women remains unknown. A potential correlation between ethnicity and radiation therapy toxicities has been suggested, with socioeconomic factors considered potential confounders [12, 13].

Therefore, we conducted a multi-institutional phase II study of ultra-HF-WBI after BCS for breast cancer in Japan (the UPBEAT study) on behalf of the Kyoto Radiation Oncology Study Group (KROSG) to evaluate the safety and efficacy of ultra-HF-WBI in Japanese patients with early breast cancer who had undergone BCS [14]. Herein, we report the results of an interim analysis of the UPBEAT study, which was conducted to assess acute adverse events (AEs) and determine the safety of proceeding with the study.

## Patients and methods

### Study design

**Fig. 1:**
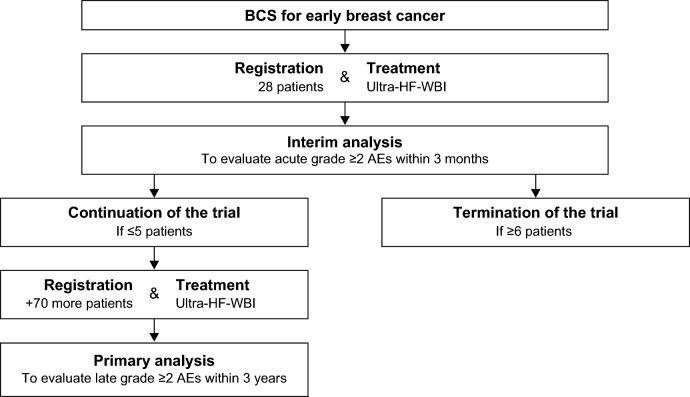
Japanese women who had previously undergone BCS were registered and received ultra-HF-WBI of 26 Gy in five fractions over one week. When the number of registered patients reached 28, patient enrollment was paused. An interim analysis was performed after three months of follow-up to determine whether it was safe to continue the study, up to a total of 98 patients. *BCS* breast-conserving surgery, *HF* hypofractionated, *WBI* whole-breast irradiation, *AE* adverse event

The UPBEAT study is a multi-institutional phase II study registered in the UMIN Clinical Trials Registry (UMIN000047080) on March 4, 2022. The protocol for this study was approved by the Institutional Review Board of Kobe City Medical Center General Hospital and has been reported previously [14]. An overview of this trial is shown in Fig. 1. Herein, we enrolled patients who had previously undergone BCS and treated them with ultra-HF-WBI as a protocol treatment. Upon enrolling 28 patients, patient registration was paused for three months. Following this, we conducted a preplanned interim analysis that evaluated AEs after three months of follow-up to determine the safety of proceeding with the trial.

### Patients

Eligible patients were required to meet all of the following criteria: no distant metastasis on any examination before surgery; had undergone BCS for breast cancer; pathological eligibility criteria, including invasive carcinoma or ductal carcinoma in situ (DCIS), pathological stage of Tis–T3 N0–N1 (including Tis Nx), and negative margin as no tumor on ink; radiation therapy initiated within 70 days of BCS, or if additional resection was performed, within 70 days of additional resection and 140 days of BCS, or within 42 days of the last day of adjuvant chemotherapy; Asian ethnicity and female sex; age ≥ 40 years at the time of registration; Eastern Cooperative Oncology Group (ECOG) performance status of 0–1; no prior radiation therapy administered to the chest except to the contralateral breast; and written informed consent provided by the patient in Japanese. The exclusion criteria are described in the previously published study protocol [14]. In the current study, all staging was based on the eighth edition of the TNM Classification of Malignant Tumours by the Union for International Cancer Control.

### Treatment

All patients underwent computed tomography (CT) simulations in the supine position, with the ipsilateral arm or both arms elevated. A patient-fixing device was mandatorily used for this purpose. For CT simulation, radiopaque markers were used to identify the boundaries of the breast. For spatial localization during each treatment, megavoltage electronic portal imaging or a similar imaging system was used. During the simulation process and each treatment session for patients with cancers of the left breast, deep inspiration breath-holding was recommended to reduce the dose delivered to the heart [15, 16]; however, this was not essential, and free breathing was permitted in all cases.

The target volumes were delineated as follows: the clinical target volume (CTV) included the whole of the conserved breast, excluding the muscle and the underlying rib cage; the planning target volume (PTV) included the CTV plus a typical margin of 5 mm; the PTV for a dose–volume histogram (PTV_DVH), defined to evaluate the PTV dose, was cropped to 5 mm inside the skin and 5 mm from the lung surface. The major organs at risk in ultra-HF-WBI were the ipsilateral lung and heart. Dosimetric criteria were established (Online Resource 1) to ensure the quality of radiation treatment planning. The use of wedge filters (including dynamic wedges) was recommended, and the field-in-field technique was permitted. The use of 4–10 MV X-rays was mandatory, whereas the use of a bolus to the skin was not permitted. Boost irradiation and supraclavicular irradiation were also not permitted.

Ultra-HF-WBI was delivered to the whole breast after BCS at a daily dose of 5.2 Gy, once per day, five times per week, and up to a total of 26 Gy in five fractions over a recommended treatment period of ≤ 10 days. Hormonal and/or anti-HER2 therapies were permitted during the treatment period; however, chemotherapy was not allowed.

### Endpoints and assessment methods

In this interim analysis, the endpoint was the rate of acute AEs of grade ≥ 2. Acute AEs were defined as toxicities experienced within 90 days of initiating radiation therapy, and were evaluated using the Common Terminology Criteria for Adverse Events (CTCAE) v5.0. The evaluation was performed immediately after treatment (within two weeks following the completion of WBI) and three months following the initiation of WBI (with an acceptable scale of one month before and after). The primary objective of our study was to determine the most frequent AEs experienced following WBI—namely, radiation dermatitis (including erythema and desquamation), pneumonitis, pain (including chest wall pain, breast pain, and skin pain), fracture, telangiectasia, superficial soft tissue fibrosis, pericarditis, and myocardial infarction.

### Statistical analysis

For the interim analysis, the required sample size was calculated to be 25 patients, with a one-sided alpha level of 10%, power of 80%, threshold value of 30%, and expected value of 12.4%, which was determined based on the proportion of acute AEs of grade ≥ 2 (38/306) reported in the JCOG0906 study [4, 5]. To account for deviations from the prescribed procedure, the total sample size in the interim analysis was estimated to be 28.

We considered that if acute AEs of grade ≥ 2 were found in ≤ 5 patients, it would represent an acceptable proportion and patient registration could be resumed. Otherwise, we would conclude that ultra-HF-WBI were markedly unsafe in Japanese women, and the study would be terminated.

## Results

In the current study, a total of 28 patients were enrolled from seven institutes between May and December 2022. Among these, 26 received ultra-HF-WBI, while 2 were excluded due to postoperative seroma infections that resulted in delayed initiation of WBI (i.e., significant deviation from the study protocol). In this interim analysis, all 26 evaluated patients survived and had no ipsilateral breast cancer relapse.

### Patient and treatment characteristics

Table 1 summarizes patient and treatment characteristics. The median age of the 28 enrolled patients was 61 years (range: 44–81). Overall, 17 patients had invasive ductal carcinoma (IDC), 8 had DCIS, and 3 had other histological types. Furthermore, 8 patients had pStage 0, 16 had pStage IA, 3 had pStage IIA, and 1 had pStage IIB. Neoadjuvant chemotherapy was administered to 5 patients, and adjuvant therapy to 19 (endocrine therapy to 17, HER2-targeted therapy to 1, and both therapies to 1).

**Table 1:** Patient and treatment characteristics (n = 28)

Characteristic	
Age, median (range) years	61 (44–81)
ECOG performance status, 0/1	28/0
Tumor site, right/left	19/9
Clinical tumor size, median (range) mm	14 (5–40)
Clinical TNM (UICC 8th)	
Tis/T1a/T1b/T1c/T2	6/2/7/10/3
N0/N1	27/1
0/IA/IB/IIA/IIB	6/19/0/2/1
Histological type, IDC/DCIS/Others	17/8/3
Surgery, BCS/BCS + SLNB	5/23
Pathological TNM (UICC 8th)	
Tis/T1a/T1b/T1c/T2	8/4/4/8/4
N0/N1a	27/1
0/IA/IB/IIA/IIB	8/16/0/3/1
Biological status, ER + /PgR + /HER2 +	25/20/4
Neoadjuvant chemotherapy	5
Adjuvant therapy	
No	9
Endocrine therapy	17
HER2-targeted therapy	1
Endocrine therapy and HER2-targeted therapy	1

*ECOG* Eastern Cooperative Oncology Group, *UICC* Union for International Cancer Control, *IDC* invasive ductal carcinoma, *DCIS* ductal carcinoma *in situ*, *BCS* breast-conserving surgery, *SLNB* sentinel lymph node biopsy, *ER* estrogen receptor, *PgR* progesterone receptor, *HER2* human epidermal growth factor receptor 2

Most patients were treated under free breathing conditions, using 4–6 MV X-ray irradiation and the field-in-field technique. The radiation treatment characteristics and dosimetric results are presented in Online Resource 2 and Online Resource 3, respectively. The median volume of the CTV, maximum dose of the body, V_90%_ of the PTV_DVH, and V_30%_ of the ipsilateral lung were 408 cc (range: 147–894), 107.7% (range: 105.0–109.8), 97.0% (range: 91.9–99.8), and 12.5% (range: 4.8–15.2), respectively. No deviations from the specified dosimetric criteria were observed.

### Acute adverse events

Table 2 presents the acute AEs (CTCAE v5.0) detected within three months of initiating WBI in the 26 evaluable patients. No AEs of grade ≥ 3 occurred. No pneumonitis of grade ≥ 2 was reported. One patient (4%) developed grade 2 radiation dermatitis (erythema). Of the 18 patients (69%) with grade 1 radiation dermatitis, erythema was recorded in all 18 (69%) and desquamation in 1 (4%). Other acute grade 1 AEs experienced were as follows: skin hyperpigmentation in 10 patients (38%), breast pain in 4 (15%), superficial soft tissue fibrosis in 3 (12%), and fatigue in 1 (4%). No other acute AEs of grade ≥ 2 were detected.

**Table 2:** Acute AEs evaluated using CTCAE v5.0 (n = 26)

AE	Grade 1	Grade 2
Radiation dermatitis	18 (69%)	1 (4%)
Erythema	18 (69%)	1 (4%)
Desquamation	1 (4%)	0
Skin hyperpigmentation	10 (38%)	0
Breast pain	4 (15%)	0
Superficial soft tissue fibrosis	3 (12%)	0
Fatigue	1 (4%)	0

*AE* adverse event, *CTCAE* Common Terminology Criteria for Adverse Events

## Discussion

This is the first clinical report of ultra-HF-WBI following BCS in Japanese women. The purpose of the present interim analysis was to evaluate acute AEs associated with ultra-HF-WBI when compared with those associated with CF- and HF-WBI in Japanese women, in order to establish whether it was safe to continue the UPBEAT study. The results met the predefined safety criteria: acute AEs of grade ≥ 2 were detected in ≤ 5 patients (radiation dermatitis of grade 2 in 1/26 patients). Pursuant to the protocol of the current study, acute toxicities associated with ultra-HF-WBI was within the acceptable limit among Japanese women. Therefore, the continuation of this study was deemed appropriate.

We compared the frequencies of acute AEs in our study with those in trials from European countries, particularly considering radiation dermatitis, the most frequent acute AE of WBI. In the FAST-Forward trial, acute skin toxicities of grade ≥ 2 (CTCAE) occurred in 36% (19/53) of the patients in the 26 Gy group (26 Gy in five fractions over one week) [8]. This high proportion of radiation dermatitis in the FAST-Forward trial may be partly attributable to the post-mastectomy bolus to the skin. Similar acute toxicity results (evaluated as the proportion of radiation dermatitis of grade ≥ 2) were reported from Belgium (16.2%, CTCAE [17]); Italy (6.7%, CTCAE [11]); and Spain (4%, Radiation Therapy Oncology Group [RTOG] [18]). In the present interim analysis, the proportion of patients with grade 2 radiation dermatitis was 4% (1/26). Other acute AEs in our study were also considered equivalent to those observed in the FAST-Forward or other European trials. Therefore, the proportion of acute AEs does not seem to differ between Caucasian and Japanese patients.

Several reports have described acute AEs associated with WBI in Japanese patients receiving other fractionation protocols. The JCOG0906 study [4, 5], another prospective trial on WBI using a novel fractionation regimen, was also conducted among Japanese women, and the patients were treated with WBI of 42.56 Gy in 16 fractions. No acute AEs of grade ≥ 3 were observed, and grade 2 radiation dermatitis was detected in 8.2% (25/306) of the patients. Osako et al. reported that radiation dermatitis of grade ≥ 2 occurred in 22% of patients treated with CF-WBI and 9% of those treated with HF-WBI [19]. Therefore, considering the results of the current interim analysis, the proportion of acute AEs did not increase when patients were treated with ultra-HF-WBI. A comparison of the biological effectiveness of different fractionation regimens using a linear-quadratic model revealed that, based on the α/β ratio of ~ 10 Gy, the biologically effective doses (BEDs) associated with acute skin reactions following CF-, HF-, and ultra-HF-WBI were approximately 60, 54, and 40 Gy, respectively, which are theoretical values calculated in accordance with a previous report [20]. The BED for acute skin reactions in ultra-HF-WBI was therefore smaller than those in CF- and HF-WBI, as were the BEDs for other acute reactions. Thus, we concluded that the acute safety was also theoretically within the acceptable limit.

We believe that ultra-HF-WBI represents a favorable treatment option for Japanese women with early breast cancer. The major benefit of this method is the reduction in patient burden in terms of treatment time. Ultra-HF-WBI requires commuting to the radiation therapy facility for only five days or a week at most, which is one-fifth of the commute for CF-WBI and approximately one-third of that needed for HF-WBI. Moreover, ultra-HF-WBI benefits radiation therapy facilities by reducing the burden on healthcare workers and assisting in the allocation of limited medical resources.

As a key limitation, it should be noted that this was only an interim analysis of the UPBEAT study, and the number of evaluable patients and length of the follow-up period were limited; therefore, long-term safety and efficacy could not be conclusively determined. At the time of primary and final analyses, 98 patients will have been enrolled, and long-term safety and efficacy will be reported upon completion of the 3- and 5-year follow-up periods. In addition, the safety of ultra-HF-WBI in Japanese women when boost irradiation is added was not evaluated in the present study, and the most appropriate boost irradiation schedule has not yet been identified. The FAST-Forward trial adopted 10 or 16 Gy in 2-Gy fractions [8, 9]; however, we assumed that a shorter schedule would be desirable. It has been reported that ultra-HF-WBI using simultaneous integrated boost (SIB) is tolerable in terms of acute toxicities [17, 18]. SIB might represent a better alternative, as it does not prolong the treatment period. Further research is required to identify the most tolerable boost schedules for Japanese women.

In conclusion, acute AEs related to ultra-HF-WBI were confirmed to be within acceptable limits for Japanese women when compared with AEs related to CF- or HF-WBI. Once the long-term safety and efficacy of this study have been verified, we believe that ultra-HF-WBI will become one of the standard fractionations for Japanese patients, given that a reduction in the overall treatment duration will benefit both patients with breast cancer and radiation therapy facilities.

### Supplementary Information

Below is the link to the electronic supplementary material.

Supplementary file1 (DOCX 22 KB)

Supplementary file2 (DOCX 19 KB)

Supplementary file3 (DOCX 23 KB)

## Declarations

### Research involving human and animals participants

Hyogo Prefectural Amagasaki General Medical Center, Japanese Red Cross Fukui Hospital, Japanese Red Cross Wakayama Medical Center, Kobe City Medical Center General Hospital, Kyoto City Hospital, Kyoto-Katsura Hospital, Kyoto Okamoto Memorial Hospital, Kyoto University Hospital, Nagahama City Hospital, Osaka Red Cross Hospital, Rakuwakai Otowa Hospital, Shiga General Hospital, and Shizuoka City Shizuoka Hospital. All procedures performed in studies involving human participants were conducted in accordance with the ethical standards of the institutional and/or national research committee and with the 1964 Declaration of Helsinki and its later amendments or comparable ethical standards.
