# Supplementary material for: Acute adverse events of ultra-hypofractionated whole-breast irradiation after breast-conserving surgery for early breast cancer in Japan: an interim analysis of the multi-institutional phase II UPBEAT study
Source: Breast Cancer. 2024 Apr 12;31(4):643–8. doi: 10.1007/s12282-024-01577-3 (PMC11194189; doi:10.1007/s12282-024-01577-3)
Supplement: Supplementary file 1 — Supplementary file1 (DOCX 22 KB) [file 12282_2024_1577_MOESM1_ESM.docx]

**Acute adverse events of ultra-hypofractionated whole-breast irradiation after breast-conserving surgery for early breast cancer in Japan: an interim analysis of the multi-institutional phase II UPBEAT study**

**Journal: *Breast Cancer***

Peter J. K. Tokuda, Takamasa Mitsuyoshi, Yuka Ono, Takahiro Kishi, Yoshiharu Negoro, Setsuko Okumura, Itaru Ikeda, Takashi Sakamoto, Yumi Kokubo, Ryo Ashida, Toshiyuki Imagumbai, Mikiko Yamashita, Hiroaki Tanabe, Sayaka Takebe, Mariko Tokiwa, Eiji Suzuki, Chikako Yamauchi, Michio Yoshimura, Takashi Mizowaki, Masaki Kokubo, and on behalf of the Kyoto Radiation Oncology Study Group

Corresponding author email: mitsu.t@kuhp.kyoto-u.ac.jp

**Dosimetric criteria of targets and organs at risk**

| **Target** |  | **Mandatory** | **Optimal** |
| --- | --- | --- | --- |
| PTV_DVH | Lower limit | V_90%_ ≥ 90% | V_90%_ ≥ 95% |
|  | Upper limit | V_105%_ ≤ 7% | V_105%_ ≤ 5% |
|  |  | V_107%_ ≤ 2% | |
|  |  | D_max_ ≤ 110% | |
| **Organ at risk** |  |  | |
| Body | Upper limit | D_max_ ≤ 110% | |
| Ipsilateral lung | Upper limit | V_30%_ ≤ 17% | V_30%_ ≤ 15% |
| Heart | Upper limit | V_25%_ ≤ 5% | |
|  |  | V_5%_ ≤ 25% | |

*PTV_DVH* planning target volume for a dose–volume histogram, *V_X%_* percentage of an organ or target volume receiving at least X% of the prescribed dose, *D_max_* maximum point dose to an organ or target in radiation therapy for cancer treatment
