# Supplementary material for: Acute adverse events of ultra-hypofractionated whole-breast irradiation after breast-conserving surgery for early breast cancer in Japan: an interim analysis of the multi-institutional phase II UPBEAT study
Source: Breast Cancer. 2024 Apr 12;31(4):643–8. doi: 10.1007/s12282-024-01577-3 (PMC11194189; doi:10.1007/s12282-024-01577-3)
Supplement: Supplementary file 2 — Supplementary file2 (DOCX 19 KB) [file 12282_2024_1577_MOESM2_ESM.docx]

**Acute adverse events of ultra-hypofractionated whole-breast irradiation after breast-conserving surgery for early breast cancer in Japan: an interim analysis of the multi-institutional phase II UPBEAT study**

**Journal: *Breast Cancer***

Peter J. K. Tokuda, Takamasa Mitsuyoshi, Yuka Ono, Takahiro Kishi, Yoshiharu Negoro, Setsuko Okumura, Itaru Ikeda, Takashi Sakamoto, Yumi Kokubo, Ryo Ashida, Toshiyuki Imagumbai, Mikiko Yamashita, Hiroaki Tanabe, Sayaka Takebe, Mariko Tokiwa, Eiji Suzuki, Chikako Yamauchi, Michio Yoshimura, Takashi Mizowaki, Masaki Kokubo, and on behalf of the Kyoto Radiation Oncology Study Group

Corresponding author email: mitsu.t@kuhp.kyoto-u.ac.jp

**Radiation treatment characteristics (n = 26)**

| **Characteristic** | | |
| --- | --- | --- |
| Breathing conditions | Free breathing | 23 |
|  | Deep inspiration breath-holding | 3 |
| Irradiation field size | Craniocaudal, median (range) cm | 20.0 (17.3–21.7) |
|  | Ventrodorsal, median (range) cm | 8.3 (6.7–11.7) |
| X-ray beam energy | 4 MV | 10 |
|  | 6 MV | 14 |
|  | 8 MV | 1 |
|  | 4 MV + 10 MV | 1 |
| Beam technique | Field-in field technique | 19 |
|  | Dynamic wedges | 5 |
|  | Physical wedges | 2 |
| Treatment duration | 5 days | 3 |
|  | 7 days | 21 |
|  | 8 days | 2 |
