# Supplementary material for: Acute adverse events of ultra-hypofractionated whole-breast irradiation after breast-conserving surgery for early breast cancer in Japan: an interim analysis of the multi-institutional phase II UPBEAT study
Source: Breast Cancer. 2024 Apr 12;31(4):643–8. doi: 10.1007/s12282-024-01577-3 (PMC11194189; doi:10.1007/s12282-024-01577-3)
Supplement: Supplementary file 3 — Supplementary file3 (DOCX 23 KB) [file 12282_2024_1577_MOESM3_ESM.docx]

**Acute adverse events of ultra-hypofractionated whole-breast irradiation after breast-conserving surgery for early breast cancer in Japan: an interim analysis of the multi-institutional phase II UPBEAT study**

**Journal: *Breast Cancer***

Peter J. K. Tokuda, Takamasa Mitsuyoshi, Yuka Ono, Takahiro Kishi, Yoshiharu Negoro, Setsuko Okumura, Itaru Ikeda, Takashi Sakamoto, Yumi Kokubo, Ryo Ashida, Toshiyuki Imagumbai, Mikiko Yamashita, Hiroaki Tanabe, Sayaka Takebe, Mariko Tokiwa, Eiji Suzuki, Chikako Yamauchi, Michio Yoshimura, Takashi Mizowaki, Masaki Kokubo, and on behalf of the Kyoto Radiation Oncology Study Group

Corresponding author email: mitsu.t@kuhp.kyoto-u.ac.jp

**Dosimetric results**

| **Target** |  | **Median** | **Range** |
| --- | --- | --- | --- |
| PTV_DVH | V_90%_ | 97.0% | (91.9–99.8) |
|  | V_105%_ | 0.8% | (0.0–6.2) |
|  | V_107%_ | 0.0% | (0.0–1.3) |
|  | D_max_ | 107.4% | (104.9–109.7) |
|  | Volume | 397 cc | (126–897) |
| CTV | Volume | 408 cc | (147–894) |
| **Organ at risk** |  |  |  |
| Body | D_max_ | 107.7% | (105.0–109.8) |
| Ipsilateral lung | V_30%_ | 12.5% | (4.8–15.2) |
| Heart (left breast cancer [n = 9]) | V_25%_ | 0.1% | (0.0–3.5) |
|  | V_5%_ | 4.0% | (1.8–14.8) |

*PTV_DVH* planning target volume for a dose–volume histogram, *CTV* clinical target volume, *V_X%_* percentage of an organ or target volume receiving at least X% of the prescribed dose, *D_max_* maximum point dose to an organ or target in radiation therapy for cancer treatment
